# Road traffic injuries in Nepal during COVID-19 lockdown

**DOI:** 10.12688/f1000research.26281.3

**Published:** 2021-02-01

**Authors:** Bhagabati Sedain, Puspa Raj Pant

**Affiliations:** 1 Department of Population Studies, Padma Kanya Multiple Campus, Tribhuvan University, Kathmandu, Bagmati, 44600, Nepal; 2 Centre for Academic Child Health, University of the West of England, Bristol, UK; 3 Nepal Injury Research Centre, Bhaktapur, Nepal

**Keywords:** COVID-19, Lockdown, Road Traffic Crashes, Injuries, Deaths

## Abstract

**Background**: As the world is busy addressing COVID-19, road traffic injuries, another major cause of death is continuously killing people on the roads. In Nepal, there were frequent media reports of occurrences of road crashes, injuries, and deaths despite nationwide lockdown. This paper aims to describe the situation of road traffic crashes and casualties during the period of complete lockdown.

**Methods**: This study used secondary data from two sources: Nepal Police and media reports between 24 March and 14 June 2020 (because the government lifted the nationwide lockdown from 15 June 2020). Available details of crashes, deaths, and injuries for this period were extracted from media reports and the summary data that was obtained from the Police.  We have included data from both sources in the results.

**Results**: Nepal Police recorded 1,801 incidents of road crashes during the 82 days of the COVID-19 lockdown with 256 deaths (on average 3.1 deaths daily) and 1,824 injuries (on average 22.2 injuries daily). Motorcycles comprised over 21% of all vehicles involved in crashes. Ambulances and other vehicles for essential services were also found to be involved in crashes. Speeding itself was the cause for almost a quarter of the incidents during the lockdown.

**Conclusions**: Although a reduction in the number of road crashes and related injuries and fatalities was observed, this reduction was not as substantial as anticipated during the heavy restrictions on vehicular movement imposed during the lockdown. Media reports were mainly found to be reporting the crashes where deaths occurred, but police records also included nonfatal injuries. The incidence of crashes in this period shows that it is important to work for road safety to save lives from road traffic crashes in Nepal.

## Background

The world experienced a series of unprecedented events since December 2019 after the detection of the Novel Coronavirus disease (COVID-19) ( Asian Development Bank, 2020). The World Health Organisation (WHO) declared it a worldwide pandemic on 11 March 2020 ( World Health Organization, 2020). During this period, social-distancing and lockdowns were implemented throughout the world. As of 14 June 2020, the spread of COVID-19 has reached all countries and territories around the globe with 282,733 deaths ( Worldometer, 2020)

The concept of restrained movement and physical distancing is believed to support the breaking of the chain of infection ( World Health Organization, 2014) and slowing the spread of the virus by limiting contact with infected people and contaminated surfaces. In many countries, everyone but essential workers have been instructed to stay at home and work from home. Consequently, transportation through all means has reduced in a never-before-seen manner. There are also reports of improvement in air quality ( Wang *et al.*, 2020) and reduced bed occupancy for road crash trauma in emergency departments ( Zhu *et al.*, 2020), which might have enabled health service systems to prepare and cope with a sudden rise in the number of COVID-19 hospitalisation. However, keeping people at home was not an easy job; governments had to impose notices with strict provisions – including fines and potential imprison if their decisions were violated.

Nepal also joined the global practice for the prevention of the spread of COVID-19 by declaring a ban on long-distance public travels from 22 March 2020 through the Prime Minister's statement to the nation. *"*All international flights coming to Nepal have been suspended effective from March 22 until 31. Effective from March 22, long-distance passenger vehicles will be suspended throughout Nepal for some time. Crowded places like cinema halls have been shut down for the time being." Prime Minister KP Sharma Oli, 20 March 2020 ** ( Embassy of Nepal, 2020).

**Figure 1.**
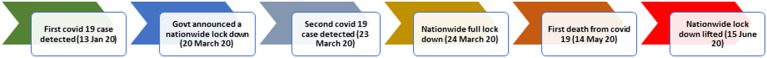
Timeline of COVID-19 related event in Nepal. Sources: Box 1 First covid case: Bastola *et al.*, 2020. Box 2 lockdown announcement: Embassy of Nepal, 2020. Box 3 Second covid 19: Dhakal & Karki, 2020. Box 4 Full Lock down: Mahato *et al.*, 2020. Box 5 first covid 19 death: Poudel, 2020. Box 6 Lockdown lifted: Kharel, 2020.

Within the window of the partial lockdown (21 March to 23 March), an estimated 1.5 million residents left the capital Kathmandu for different parts of the country. Similarly, about half a million migrant workers from India also returned to their homes in the wake of the government’s decision to lock down the country ( Pokhrel & Awale, 2020). This resulted in a sudden rise in the use of motorised vehicles during the 21st, 22nd, and 23rd of March. Meanwhile, the second case of COVID-19 was detected on 23 March ( Figure 1). Only then did the Government decided to impose a countrywide complete lockdown, from 24 March 2020 ( Figure 1) ( Budhathoki, 2020). Hence, the country's efforts and resources converged towards the prevention of coronavirus transmission.

However, the government authorised a special pass-permit to use private vehicles and motorcycles in case of an emergency. Only vehicles required for essential services, i.e. ambulances, police, fire service, milk-tankers, water-tankers, and food deliveries, were allowed on the road without the pass. Due to these activities, a sudden decline in vehicular movement was observed in Nepal. Subsequently, a large reduction in the number of crashes and casualty was expected during this lockdown. Unfortunately, there were frequent media reports of road crashes, injuries, and deaths despite the nationwide lockdown.

This paper aims to describe the situation of road traffic crashes and the subsequent casualties during the period of COVID-19 lockdown using secondary sources of data. In the study, we have presented the results from the two data sources to provide maximum possible details complemented by one-another.

## Methods

This study utilised two secondary data sources, i.e. media reports, and published or unpublished police records. Data collection was done in two ways: a daily online search of media reports for vehicle crash incidents on Google, which was done using search terms in the Nepali language in order to capture most of the reports across the country. The search terms were “deaths or injuries”, “road crash”, “car crash”, “motorcycle crash”, “vehicle crash”, “pedestrian hit by”, “bicyclist hit by”, “ambulance crash”, “tractor crash”, “truck crashes”, or “crashes or collisions occurred during lockdown;”. Similarly, data was also collected by contacting the police to obtain road crash records. From both these data sources, only limited number of variables could be extracted. The location of crash, the vehicles, animals, people, or objects involved, the resultant number of deaths & injuries, and the age and gender of victims were extracted from media reports into an Excel spreadsheet. The total number of crashes, deaths and injuries occurring in districts and provinces were taken from police records. This paper includes the road crash information for 82 days of the lockdown (24 March to 14 June 2020) from media reports and police records. The exact location and types of vehicles involved in fatal crashes were not available from the Police data, therefore the exact details of the vehicles and the location of crashes were extracted from the media reporting. With the available information on fatal crashes for *Palika* level (local government unit), the cases were nationally mapped for these units. In this study, we have presented the results from the two data sources to provide maximum possible details complemented by one another.

## Results

Altogether, there were 1,801 incidents of road crashes recorded by the traffic police in 82 days (24 March to 14 June 2020) of lockdown from all seven provinces of Nepal, which included 2,602 vehicles (96% motorized) that claimed 256 lives and led to a further 1,824 injuries (among which 32% were severely injured). However, the media mostly reported fatal crashes, as 200 deaths and 322 injuries were extracted through media reports for the same period. The number of deaths and injuries reported by local media and taken from police records are given in the Underlying data ( Sedain & Pant, 2020).

In this lockdown, no vehicles were allowed to operate without a government-issued pass for essential services. Police records show that in 82 days of full lockdown, an average of 3.1 people died and 22.2 people were injured daily as a result of road crashes. The media reporting of fatal road crashes was 21.8% less than the police record, and very few injuries and vehicle crashes were reported ( Table 1).

**Table 1.** Distribution of the road crash incidents, vehicles involved in crashes, deaths and injuries during national level COVID-19, 82 days lockdown from police record and media reporting in Nepal.

Places	Vehicle involved in crashes	Incidents	Deaths	Injuries
Province 1	179 (6.9%)	137 (7.6%)	36 (14.1%)	186 (10.2%)
Province 2	337 (13.0%)	261 (14.5%)	44 (17.2%)	332 (18.2%)
Bagmati	130 (5.0%)	112 (6.2%)	26 (10.2%)	186 (10.2%)
Gandaki	58 (2.2%)	57 (3.2%)	15 (5.9%)	70 (3.8%)
Lumbini	308 (11.8%)	289 (16.0%)	68 (26.6%)	359 (19.7%)
Karnali	27 (1.0%)	25 (1.4%)	15 (5.9%)	50 (2.7%)
Sudurpaschim	66 (2.5%)	45 (2.5%)	25 (9.8%)	61 (3.3%)
Kathmandu Valley	1497 (57.5%)	875 (48.6%)	27 (10.5%)	580 (31.8%)
**Total of** **Nepal Police**	**2602 (100%)**	**1801(100%)**	**256 (100%)**	**1824(100%)**
***Daily average***	***31.7 vehicles***	***22.0 incidents***	***3.1 deaths***	***22.2 injuries***
**Media** **reported**	**544**	**272**	**200**	**322**

*Source: Nepal Police Province 1, 2, Bagmati, Gandaki, Lumbini, Karnali and Sudurpaschim headquarters, Metropolitan Traffic Police Office and media reports of road crashes reported*

Kathmandu Valley comprises of three districts, namely Kathmandu, Bhaktapur, and Lalitpur. Nepal Police has not recorded the road traffic deaths separately for these three districts and the records of the crashes were presented for Kathmandu Valley as a whole. Therefore, by including the three districts of Kathmandu Valley, Table 2 displays road traffic deaths from 12 districts. In the lockdown period, these 12 districts accounted for more than half (53.4%) of the total deaths in Nepal. The largest number of people were killed in Kathmandu Valley’s roads, followed by Kailali. Furthermore, Lumbini Province has the highest proportion (20.3%) of road traffic deaths, followed by Bagmati Province (13.6%).

**Table 2.** Districts with highest number of road traffic deaths in Nepal during the COVID-19 lockdown.

District	Province	Number of deaths	Percentage
Kathmandu Valley *	Bagmati	27	10.5
Kailali	Sudurpaschim	20	7.8
Banke	Lumbini	19	7.4
Nawalparasi West	Lumbini	13	5.1
Morang	Province 1	11	4.3
Siraha	Province 2	11	4.3
Dang	Lumbini	11	4.3
Rautahat	Province 2	9	3.5
Rupandehi	Lumbini	9	3.5
Sindhuli	Bagmati	8	3.1
Subtotal of deaths in 12 districts		138	53.9
Total deaths in Nepal		256	100.0

Source: Nepal Police Province 1, 2, Bagmati, Lumbini and Sudurpaschim headquarters record for road crashes incidents. *Kathmandu Valley comprise three districts (Kathmandu, Bhaktapur and Lalitpur)

**Figure 2.**
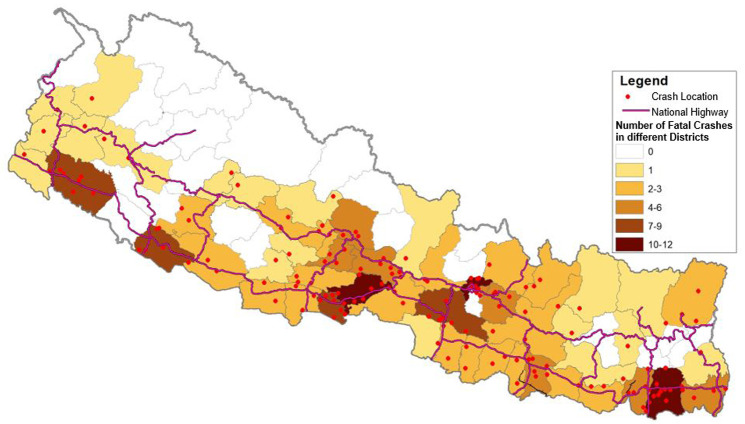
Visualization of road crashes deaths during COVID-19 lockdown reported in the media by Palika and national highways. Source: Locations of road crashes and fatalities from media reports. Map reproduced with the permission of the Survey Department of Nepal (2020) ( Nepal Government Survey Department, 2020).

The information on fatal crashes by location extracted from media reports has been visualized ( Figure 2) to show the crash-prone areas of Nepal. The visualization has shown that fatal crashes were concentrated more in the middle and lower region of country. Regarding provinces, the fatal crashes were higher in various locations of Bagmati Province and Lumbini Province. The visualization additionally demonstrates that a large number of fatal crashes have occurred in local units in the junction of national highways of different local government units.

Table 3 shows the type of vehicles involved in crashes and the objects, animals or people they collided with. Motorcycles were the most common vehicles involved in fatal crashes, as usual. Among the total vehicles involved in the crashes, more than one-fifth (22.1%) were motorcycles followed by jeeps, tractors, and trucks. Along with other vehicles, 20 ambulances were found to be involved in road crashes which either hit other vehicles, people, animals or roadside objects. The majority of the vehicles involved in the crashes were reportedly out of the driver's control (52.2%) due to speeding. Similarly, 37 pedestrians were hit by vehicles in these 82 days. Motorcyclists, pedestrians and cyclists were the most at-risk road users during road users from motorcyclists and the vehicles at essential services i.e. jeep/cars, tractors, trucks and ambulances ( Table 3).

**Table 3.** Distribution of road crashes during national level COVID-19 lockdown in Nepal according to the types of vehicles involved and their counterparts. Source: Media reporting of road crashes for the lockdown period (24 March to 14 June, 2020).

Vehicles involved			Counterparts		
Type	Number	Percent	Type	Number	Percent
Ambulance	20	7.4	Animal	1	0.4
Cyclist	3	1.1	Auto tempo	1	0.4
Bus	10	3.7	Cyclist	9	3.3
Excavator	1	0.4	Car	3	1.1
HDV	1	0.4	Jeep	4	1.5
Jeep	54	19.9	Motorcycle	40	14.7
Motorcycle	76	27.9	Lamppost	3	1.1
Power trailer	2	0.7	Pedestrian	37	13.6
Tanker	2	0.7	Tractor	4	1.5
Tipper	10	3.7	Tanker	1	0.4
Tractor	52	19.1	Tree	2	0.7
Truck	34	12.5	Truck	6	2.2
Unidentified	5	1.8	None (Uncontrolled *)	142	52.2
Van	2	0.7	Unidentified	19	7.0
**Total**	**272**	**100.0**	**Total**	**272**	**100.0**

*'uncontrolled' is the term when it is reported 'the vehicle went beyond the control of the driver'

**Figure 3.**
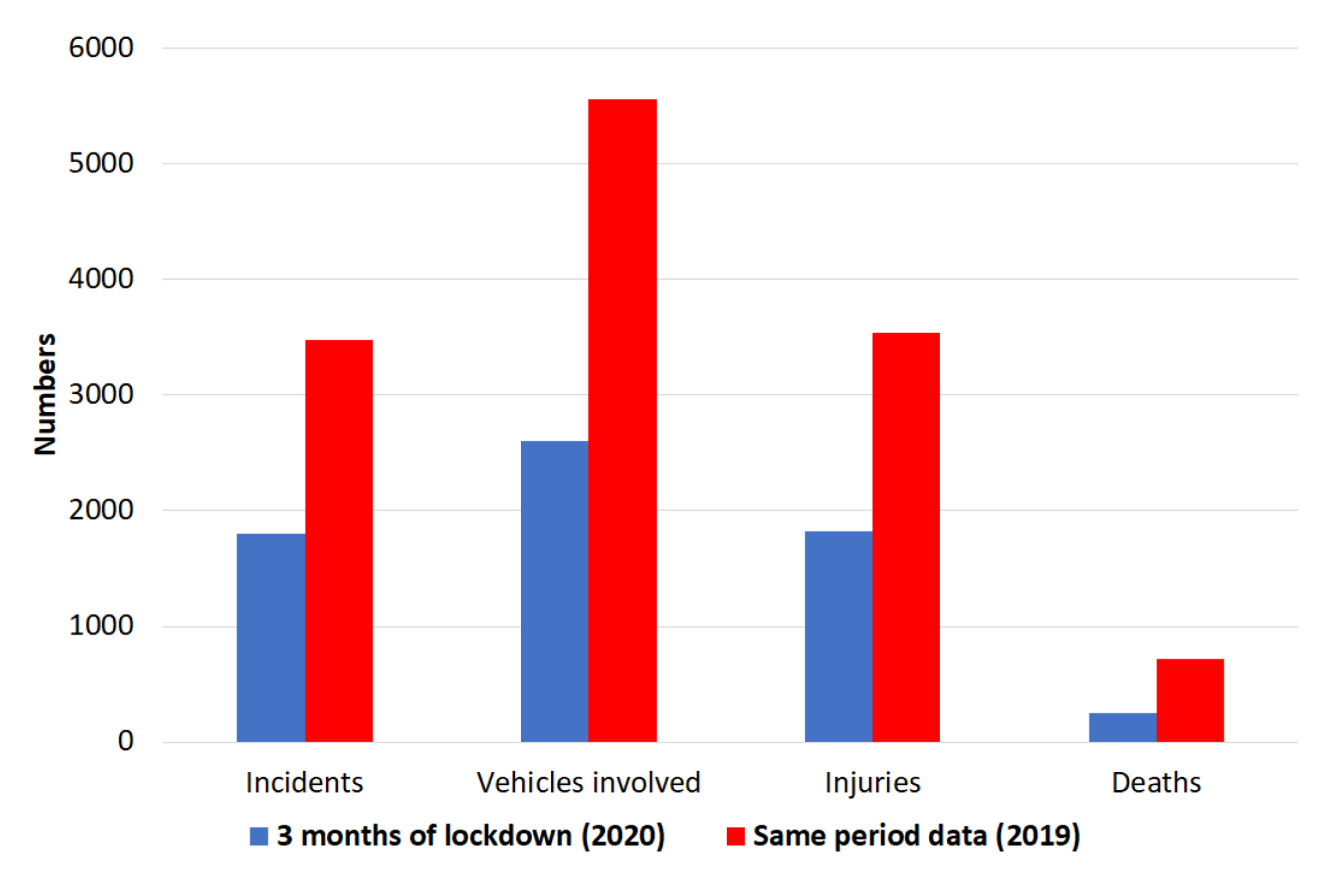
Comparison of incidents of road crashes, injuries and deaths during the 3 months of COVID-19 lockdown and the same period in 2019.

Comparing the police data for lockdown and same period last year shows a considerable reduction in the number of incidents, involved vehicles and casualties ( Figure 3). Given the lower number vehicles allowed to operate the figures are still high.

## Discussion

Since testing for COVID-19 cases commenced in Nepal, 5,760 positive cases (as of 14 June 2020) and 19 deaths have been identified ( Worldometer, 2020). Meanwhile, 256 deaths and 1,824 injuries from road crashes were recorded between 24 March to 14 June 2020.

Road traffic injuries are the leading cause of death for the people aged 5–29 years worldwide ( World Health Organization, 2018). It is also the leading cause of death and disabilities among people aged between 15 and 49 years in Nepal ( Pant *et al.*, 2020). Regardless of the figures, which may vary from source to source, we aim to highlight road safety measures and their importance for the essential-service vehicles during adversity.

The total burden of road traffic injuries in Nepal is calculated to be approximately 123 million USD for 2017 ( Banstola *et al*., 2020). The amount of indirect cost of road traffic injuries ranges from 51% in Iran to 90% in Nepal ( Banstola *et al*., 2020; Rezaei *et al*., 2014). The toll of road crash deaths has demonstrated the economic impact of road crashes in Nepal. Two-wheeled motorized vehicles (motorcycles and scooters) were most frequently involved in crashes and are found to be putting the largest burden on the economy directly and indirectly ( Sapkota *et al.*, 2016). Tractors and jeeps were the second-most frequently involved vehicles in road crashes, which is shown by both the police and media records. An incident of injury tends to become a matter of interest to the media even in an adverse situation. Therefore, not all incidents of road crashes are covered by media. From our data, it is also apparent that the fatal cases are consistently reported in police records and media reports but cases of injuries are much less reported by the media.

From police records, an average of 154 incidents of road crashes took place weekly, killing 22 people and causing 156 injuries during the period of lockdown. In the normal situation, 7.6 people die in the road crashes ( Nepal Police, 2019) and whereas in this lockdown even with the minimal transport mobility, on average 3.1 people died per day in Nepal. Comparing the police data for lockdown and same period in previous years, it shows considerable reduction in the number of incidents, involved vehicles and casualties ( Figure 3). Given the lower traffic volume during lockdown, these figures can be considered to be high. Similarly, the ratio of deaths and injuries has spiked (in Kathmandu valley) from 1 death per 46.3 injuries during the non-lockdown period ((Police Records 2019), to 1 death per 20.6 injuries in lockdown. Nationally, the number of casualty (deaths and injuries combined) per 100 vehicles (involved in road crashes) was 79.9 compared to 76.5 the same period last year. Perhaps the injured individuals were involved in more severe crashes during lockdown due to people's tendency to maintain higher speeds on the road. Drivers want to drive their vehicles at high speeds for different reasons ( Gabany *et al.*, 1997), and when the roads are empty, speeding might become obvious if there are no measures in place for speed control.

### What do the data indicate?

The casualty data indicate that the burden of road crashes remains high in the lockdown period, a discovery that is different from a popular belief that causality or crashes have decreased substantially. In the absence of evidence-based practice of road safety, people incorrectly assume that reduced vehicular movement automatically reduces the risks of a crash. Given the small number of vehicles in operation, the problem is rather big. This increased number of deaths and injuries during the lockdown in Nepal can be related to higher speed due to lower traffic volume and limited law enforcement. High speed means higher impact if there is a crash. It has also reported elsewhere that speed law violations and failing to stop due to high speeding were increased during lockdown ( Inada *et al.*, 2021).

These crash and casualty figures worryingly indicating the magnitude of the problem when regular transportation will eventually resume in Nepal. In rural areas, the use of tractors on unsafe roads increases the risks of crashes. Further, our findings also indicate the lack of a safety culture among the operators of the essential services (including ambulances and the vehicles used by law enforcers). The current focus of the government is to improve roads, but free roads encourage drivers to speed, which is dangerous in terms of road crashes. Therefore, a system of speed monitoring must also be integrated.

## Conclusion

Roadways are the major means of transport in Nepal. Just before this lockdown, a large number of people had to make journeys to their homes (mainly going out of Kathmandu and coming to Nepal from bordering India) by roads; many of those were exposed to the risks of unsafe roads transportation which led to their deaths and injuries. Our study found that deaths on Nepali roads was not stopped during lockdown. Comparing the pattern of road crashes during the same period last year, lockdown witnessed almost half of the number of incidents (1,801 vs 3,480) and the number of vehicles involved in crashes (2,602 vs 5,560). When comparing the statistics with the situation with the three months before the lockdown, it was observed that the percentages of tractors, trucks/tankers and cyclists was higher (published police records). In cities, traffic congestion was eased during the lockdown which consequently resulted higher speed, increasing the chances and impact of crashes. Therefore, awareness of safety and taking into account road and weather conditions when deciding to take a journey would help to keep people safe on the roads. Therefore, this lockdown has reinforced how important the management of safer mobility issue is in Nepal. Interestingly, some of the preventative measures that have been proven effective to decelerate the spread of coronavirus apply in the context of road safety as these measures can teach us something for the road safety epidemic as well ( Job, 2020). The Government of Nepal has mobilised unprecedented amount of resources in terms of human resources, budget and materials to address COVID-19 which has kept the rates of infection and deaths at minimum. If similar efforts and investments are done to address the problem of road traffic injuries, it would be possible to reverse the trend of ever-increasing burden of road injuries.

## Data availability

### Underlying data

Figshare: Road Traffic Injuries in Nepal during COVID-19 Lockdown_ Media reporting and Police record (24 March to 14 June, 2020).csv. https://doi.org/10.6084/m9.figshare.12958373.v3 ( Sedain & Pant, 2020).

This project contains the following underlying data:

- Road Traffic Injuries in Nepal during COVID-19 Lockdown_Media reporting (24 March to 14 June, 2020).csv. (Road traffic injuries and deaths reported by local media.)
- Road Traffic Injuries in Nepal during COVID-19 Lockdown_Police records (24 March to 14 June, 2020).xlsx.csv. (Road traffic injuries and deaths taken form police records.)

Data are available under the terms of the Creative Commons Attribution 4.0 International license (CC-BY 4.0).
