# Supplementary material for: Road traffic injuries in Nepal during COVID-19 lockdown
Source: F1000Res. 2021 Feb 1;9:1209. Originally published 2020 Oct 8. [Version 3] doi: 10.12688/f1000research.26281.3 (PMC7818237; doi:10.12688/f1000research.26281.3)
Supplement: Peer review history

# Peer review: Reviewer response for version 3

**DOI:** 10.5256/f1000research.54194.r78581

**Published:** 2021-02-02

**Authors:** Felix Wilhelm Siebert

**Affiliations:** 1 Friedrich Schiller University Jena, Jena, Germany

No further comments.

Is the work clearly and accurately presented and does it cite the current literature?

Yes

If applicable, is the statistical analysis and its interpretation appropriate?

Partly

Are all the source data underlying the results available to ensure full reproducibility?

Yes

Is the study design appropriate and is the work technically sound?

Partly

Are the conclusions drawn adequately supported by the results?

Partly

Are sufficient details of methods and analysis provided to allow replication by others?

Yes

Reviewer Expertise:

Traffic psychology

I confirm that I have read this submission and believe that I have an appropriate level of expertise to confirm that it is of an acceptable scientific standard.

# Peer review: Reviewer response for version 2

**DOI:** 10.5256/f1000research.31067.r76068

**Published:** 2021-01-20

**Authors:** Felix Wilhelm Siebert

**Affiliations:** 1 Friedrich Schiller University Jena, Jena, Germany

The authors have addressed my major and minor comments from the first revision. Only two minor comments remain:

- For Figure 3, the authors should add a y-axis label and should consider relabeling the bars as "3 months of lockdown" and "prior year data (2019)". Potentially the Figure description should be revised to "Comparison of incidents of road crashes, injuries and deaths during the 3 months COVID-19 lockdown and the same time frame in the year before".
- Figure 3 indicates that there is indeed a decrease in the number of incidents, injuries and deaths during the lockdown in comparison to the same time frame in 2019. Hence, I would suggest to slightly reword the abstract, to more clearly state that a reduction was found. This could be done by changing the order of the first sentence of the conclusion, along the lines of: "While a reduction in the number of road crashes and related injuries and fatalities was found, this reduction was not as substantial as could have been expected due to the heavy movement restrictions imposed during the lock down". (just a suggestion).

Is the work clearly and accurately presented and does it cite the current literature?

Yes

If applicable, is the statistical analysis and its interpretation appropriate?

Partly

Are all the source data underlying the results available to ensure full reproducibility?

Yes

Is the study design appropriate and is the work technically sound?

Partly

Are the conclusions drawn adequately supported by the results?

Partly

Are sufficient details of methods and analysis provided to allow replication by others?

Yes

Reviewer Expertise:

Traffic psychology

I confirm that I have read this submission and believe that I have an appropriate level of expertise to confirm that it is of an acceptable scientific standard.

# Peer review: Reviewer response for version 2

**DOI:** 10.5256/f1000research.31067.r76067

**Published:** 2021-01-18

**Authors:** Kulanthayan KC Mani

**Affiliations:** 1 Safe Kids Malaysia, Department of Community Health, Faculty of Medicine and Health Sciences, University Putra Malaysia (UPM), Serdang, Malaysia

The authors have clarified the concerns raised and understanding the limitation during the pandemic, the best available data on RTC is to be obtained from media source compared to police data. With this statement stated in the article, the article is fine. Thanks for considering the points raised. Best wishes.

Is the work clearly and accurately presented and does it cite the current literature?

Yes

If applicable, is the statistical analysis and its interpretation appropriate?

No

Are all the source data underlying the results available to ensure full reproducibility?

Partly

Is the study design appropriate and is the work technically sound?

Partly

Are the conclusions drawn adequately supported by the results?

No

Are sufficient details of methods and analysis provided to allow replication by others?

Partly

Reviewer Expertise:

Injury prevention and safety promotion

I confirm that I have read this submission and believe that I have an appropriate level of expertise to confirm that it is of an acceptable scientific standard.

# Peer review: Reviewer response for version 1

**DOI:** 10.5256/f1000research.29008.r72705

**Published:** 2020-11-10

**Authors:** Felix Wilhelm Siebert

**Affiliations:** 1 Friedrich Schiller University Jena, Jena, Germany

The study presents secondary source road traffic injury and fatality data from Nepal for the time frame of the recent COVID-19 related lockdown between March and June 2020.

 I understood the study as follows: The authors used Nepal Traffic Police data to extract numbers of fatalities and injuries (distinguished between heavy and non-heavy injuries) for the timeframe between March and June 2020. In addition, news articles were analyzed and reports on injuries and fatalities were collected for the same timeframe. Both datasets are then compared, and the authors find that not all fatal crashes are reported on, while even less of a percentage of crashes that “only” result in injuries are reported in the news. The authors list potential factors related to accident causes and hypothesize that speeding might play a larger role in crashes during the lockdown, as the road system is less crowded.

 The article is very easy to read and all data is presented in a concise and clear way. I have two major comments before going through the manuscript in detail.

**Comparison to pre-lockdown data**

 Before reading the manuscript, I expected that the authors would compare injury and fatality data during the lockdown to data collected outside of the lockdown period, e.g. to the same days in 2019. This would give an indication if the lockdown has led to a general increase in fatalities and injuries (this would be expected). The authors report non-lockdown data in the discussion section of the article (p.5 “In the normal situation 7.6 people die in the road crashes […]“) but I would have expected this comparison sooner, i.e. in the results section.

 In addition, the comparison of the ratio of injuries to deaths should be presented sooner than in the discussion. The ratio named for the non-lockdown period (“Similarly, the ratio of deaths and injuries - a number that has surged from 1 death per 22.8 injuries during the non-lockdown period (Karkee & Lee, 2016), to 1 death per 7.1 injuries in lockdown.”) is old, as the data used in the Karkee & Lee (2016) paper is from 2013.

 Furthermore, it’s unclear if the correct data from the Karkee and Lee paper is used to arrive at the death/injury ratio. It appears that the ratio of 1 death per 22.8 injuries is calculated from Table 1 in the cited article, but erroneously only injury and fatality data for Kathmandu is used. Calculating the death/injury ratio from the correct column (listing the “total” number for the last year of available data) results in a death/injury ratio of 6.6. Hence, I advise that the authors either make it more clear which data is used for the calculation of the ratio, or (preferably) calculate the more current ratio for the whole year 2019 and for the exact same time frame as the 2020 lockdown, using 2019 data.

**Timely distribution of fatalities and injuries within the lockdown time frame**

 The authors do a great job in describing outside factors that have influenced traffic density during the lockdown, i.e. especially before the lockdown there seems to have been higher traffic as people left for their home villages / home countries. While reading, I was wondering if this potential uneven distribution of traffic within the observed time frame would be visible in the distribution of injuries and fatalities, e.g. more injuries and fatalities in the beginning of the lockdown than in the middle/end. This could be visualized easily by plotting the percentage of registered injuries and fatalities over the lockdown time frame. This could also be presented alongside the same data for the same time frame in 2019.

**Other comments:**

- The title omits the analysis of fatalities during the lockdown, the authors should consider revising the title “Road traffic injuries and fatalities during […]”
- I understand the urge to contrast numbers of road related fatalities with COVID-19 numbers, but they are very different things, mainly as they have completely different potential for exponential increases. Hence, I would suggest the authors revise the first sentence of the abstract to make road safety and COVID less contrastive.
- The results section of the abstract should present comparative data to the same time frame from a non-lockdown period.
- The conclusion part of the abstract is not supported by the results part of the abstract. The sentence “Even when the movement restrictions were imposed in Nepal, the number of road crashes was not substantially reduced.” needs to be supported by additional analyses in the manuscript, which will need to be mentioned in the results part of the abstract.
- “Within the window of the partial lockdown” please list clear time frames (i.e. dates).
- “Meanwhile, the second case of COVID-19 was detected on 23 March.” when was the first case detected? The authors should consider adding a timeline of COVID-related events in Nepal (e.g. First case, partial lockdown, lockdown, opening up, analyzed time frame).
- Do the authors have any information on the number of special “pass-permits” handed out during the lockdown? This could be related to the number of registered vehicles in Nepal, to get an idea of how much the traffic was reduced through the lockdown (hypothetically).
- In the discussion section, I would suggest the authors do not directly contrast COVID-19 and road related fatalities (same argument as before). It might be enough to change “On the contrary […]” to a different wording.
- The second and third section in the discussion (“Road traffic injuries are the leading cause of death for […]”) are quite general and do not directly relate to the results found in the study. I think they are better suited to frame road safety challenges (global and in Nepal), and would be better placed in the introduction.
- For the sentence “Similarly, the ratio of deaths and injuries - a number that has surged from 1 death per 22.8 injuries during the non-lockdown period (Karkee & Lee, 2016), to 1 death per 7.1 injuries in lockdown.” Please revise for grammar. (and please also see my first major comment above).
- The section “What do the data indicate? The casualty data indicate that the burden of road crashes remains high in the lockdown period […]” is not well supported by the results section of the paper (see my first major comment).
- In the conclusion, the authors write “Roadways are the major means of transport in Nepal. In this lockdown, large number of people had to make journeys to their homes by roads […]”. But the authors earlier state, that the main movement of people happened during partial lockdown, i.e. before the time frame analyzed in this study. One could assume that during the early days of lockdown, some people used their vehicles illegally (without the special pass-permit), but this would need to be stated more clearly. The authors should clear this up (see also my second major comment).

Is the work clearly and accurately presented and does it cite the current literature?

Yes

If applicable, is the statistical analysis and its interpretation appropriate?

Partly

Are all the source data underlying the results available to ensure full reproducibility?

Yes

Is the study design appropriate and is the work technically sound?

Partly

Are the conclusions drawn adequately supported by the results?

Partly

Are sufficient details of methods and analysis provided to allow replication by others?

Yes

Reviewer Expertise:

Traffic psychology

I confirm that I have read this submission and believe that I have an appropriate level of expertise to confirm that it is of an acceptable scientific standard, however I have significant reservations, as outlined above.

# Response

**Published:** 2020-11-26

**Authors:** Bhagabati Sedain

**Affiliations:** Tribhuvan University, Nepal

**Comparison to pre-lockdown data**

 Before reading the manuscript, I expected that the authors would compare injury and fatality data during the lockdown to data collected outside of the lockdown period, e.g. to the same days in 2019. This would give an indication if the lockdown has led to a general increase in fatalities and injuries (this would be expected). The authors report non-lockdown data in the discussion section of the article (p.5 “In the normal situation 7.6 people die in the road crashes […]“) but I would have expected this comparison sooner, i.e. in the results section.

 In addition, the comparison of the ratio of injuries to deaths should be presented sooner than in the discussion. The ratio named for the non-lockdown period (“Similarly, the ratio of deaths and injuries - a number that has surged from 1 death per 22.8 injuries during the non-lockdown period (Karkee & Lee, 2016), to 1 death per 7.1 injuries in lockdown.”) is old, as the data used in the Karkee & Lee (2016) paper is from 2013.

 Furthermore, it’s unclear if the correct data from the Karkee and Lee paper is used to arrive at the death/injury ratio. It appears that the ratio of 1 death per 22.8 injuries is calculated from Table 1 in the cited article, but erroneously only injury and fatality data for Kathmandu is used. Calculating the death/injury ratio from the correct column (listing the “total” number for the last year of available data) results in a death/injury ratio of 6.6. Hence, I advise that the authors either make it more clear which data is used for the calculation of the ratio, or (preferably) calculate the more current ratio for the whole year 2019 and for the exact same time frame as the 2020 lockdown, using 2019 data.

***Authors' response: **Thanks for cogent observations. We have addressed them as below:*

- *compare injury and fatality data during the lockdown to data collected outside of the lockdown period, e.g. to the same days in 2019. *
- *used them accordingly. We have now replaced the terms relating to 'comparison' throughout the text.*

  The ratio of deaths and injuries for lockdown and non-lockdown period

***Authors' response: **Thank you very much for spotting the error. We appreciate your advice for using more recent figures. We compared the figures with those during the same period last year (i.e. 3 months of lockdown compared with the same 3 months last year). we have revised this statement as: (“Similarly, the ratio of deaths and injuries has surged (in Kathmandu) from 1 death per 46.3 injuries during the non-lockdown period (Police Records), to 1 death per 20.6 injuries during the lockdown.” A new sentence is added "Nationally, t* *he number of casualty (death plus injuries) per 100 involved vehicles were slightly higher (79.9) during lockdown compared to the same period last year (76.5)."*

 Timely distribution of fatalities and injuries within the lockdown time frame

 The authors do a great job in describing outside factors that have influenced traffic density during the lockdown, i.e. especially before the lockdown there seems to have been higher traffic as people left for their home villages / home countries. While reading, I was wondering if this potential uneven distribution of traffic within the observed time frame would be visible in the distribution of injuries and fatalities, e.g. more injuries and fatalities in the beginning of the lockdown than in the middle/end. This could be visualized easily by plotting the percentage of registered injuries and fatalities over the lockdown time frame. This could also be presented alongside the same data for the same time frame in 2019.

**Other comments:**

 The title omits the analysis of fatalities during the lockdown, the authors should consider revising the title “Road traffic injuries and fatalities during […]”

***Authors' Response:**Thank you very much for your suggestion. We think the title is ok.*

**

 I understand the urge to contrast numbers of road related fatalities with COVID-19 numbers, but they are very different things, mainly as they have completely different potential for exponential increases. Hence, I would suggest the authors revise the first sentence of the abstract to make road safety and COVID less contrastive.

***Authors' response: **Thanks for your suggestion but we think this statement is reasonable and we would like to keep it as is.*

**

 The results section of the abstract should present comparative data to the same time frame from a non-lockdown period.

***Authors' response:** We agree that adding a figure with the information during non-lock down would enhance the text. Figure 3 has been added to the discussion section and cited in the fourth paragraph (p5). "Comparing the police data for lockdown and the same period last year shows* a *considerable reduction in the number of incidents, involved vehicles and casualties* *(Figure 3 ). Given the lower number of vehicles allowed to operate the figures are high.* *"* 

 The conclusion part of the abstract is not supported by the results part of the abstract. The sentence “Even when the movement restrictions were imposed in Nepal, the number of road crashes was not substantially reduced.” needs to be supported by additional analyses in the manuscript, which will need to be mentioned in the results part of the abstract.

***Authors Response:** We have revised the conclusion, it was also suggested by reviewer 1 (please see the conclusion section in the text).*

**

 “Within the window of the partial lockdown” please list clear time frames (i.e. dates *).*

***Authors Response: **21 march to 23 march*

 “Meanwhile, the second case of COVID-19 was detected on 23 March.” when was the first case detected? The authors should consider adding a timeline of COVID-related events in Nepal (e.g. First case, partial lockdown, lockdown, opening up, analyzed time frame).

***Authors' response: **The first case was confirmed on 13 ^th^ January and we have added a figure (Figure 1) in the introduction*

 Do the authors have any information on the number of special “pass-permits” handed out during the lockdown? This could be related to the number of registered vehicles in Nepal, to get an idea of how much the traffic was reduced through the lockdown (hypothetically).

***Authors' response**: unfortunately, no information available in this regard*

**

 In the discussion section, I would suggest the authors do not directly contrast COVID-19 and road related fatalities (same argument as before). It might be enough to change “On the contrary […]” to a different wording.

***Authors' response**: we have changed "on the contrary", as "meanwhile"*

**

 The second and third section in the discussion (“Road traffic injuries are the leading cause of death for […]”) are quite general and do not directly relate to the results found in the study. I think they are better suited to frame road safety challenges (global and in Nepal), and would be better placed in the introduction.

***Au thors'**** response:** Thanks for the suggestion, but we think these two statements are setting the context for discussion.*

 For the sentence “Similarly, the ratio of deaths and injuries - a number that has surged from 1 death per 22.8 injuries during the non-lockdown period (Karkee & Lee, 2016), to 1 death per 7.1 injuries in lockdown.” Please revise for grammar. (and please also see my first major comment above).

***Authors' response**: we have responded to it above*

**

 The section “What do the data indicate? The casualty data indicate that the burden of road crashes remains high in the lockdown period […]” is not well supported by the results section of the paper (see my first major comment).

***Authors' response:******Thanks for the suggestion, we have now revised the sentence: "The casualty data indicate that the burden of road crashes remains high in the lockdown period despite of very much restricted vehicular movement"*

  In the conclusion, the authors write “Roadways are the major means of transport in Nepal. In this lockdown, large number of people had to make journeys to their homes by roads […]”. But the authors earlier state, that the main movement of people happened during the partial lockdown, i.e. before the time frame analyzed in this study. One could assume that during the early days of lockdown, some people used their vehicles illegally (without the special pass-permit), but this would need to be stated more clearly. The authors should clear this up (see also my second major comment). 

***Authors' response:** Thanks for the suggestion, we have now revised the sentence as “Roadways are the major means of transport in Nepal. Just before this lockdown, a large number of people had to make journeys to their homes* *(mainly going out of Kathmandu and coming to Nepal from bordering India)* *by roads immediately after the announcement of the lockdown”.*

# Peer review: Reviewer response for version 1

**DOI:** 10.5256/f1000research.29008.r72710

**Published:** 2020-10-29

**Authors:** Kulanthayan KC Mani

**Affiliations:** 1 Safe Kids Malaysia, Department of Community Health, Faculty of Medicine and Health Sciences, University Putra Malaysia (UPM), Serdang, Malaysia

1. Abstract – For the methods sections, usage of secondary data from Police is fine and acceptable, but not from the media reports. Media reports does not cover all crashes and there could also be duplication of a single case is being reported by various media. Also it’s the reporter and editor view. No one to verify it. Not recommended for scientific article unless no other options.
2. Methods – is it possible to do away with the media reports and completely rely on the police data (secondary data)? What kind of information we can get from police? If that is sufficient, then go ahead with police data only.
3. Results. Table 1. The paragraph tend to indicate as though the article is trying to compare two reporting system police vs media which is not the case as I believe you are using media to complement and close the gap for info not available from police source. Intention good, however the mechanism to address it is questionable since its unverified and its individually done by reporters.
4. Table 2. Purpose of it? My understanding is based on the title, there could be two options of story flow: 1. Before and during covid: RTC and RTI status. Option 2: study RTC and RTI during covid (there must be a purpose and benefit of doing one). Till this stage not clear yet.
5. Figure 1. Purpose of it? Does it tell us during covid the mapping of RTC and RTI are different compared to non-covid time. Therefore this are the areas to be targeted and focused by the government towards addressing the problem. Is this what you intend to?
6. Table 3. Purpose of it? Any different on its distribution / patterns from normal day which warrants attention? How best can we use this info towards our next move in terms of strategies or policy change with an aim to reduce RTC and RTI during covid.
7. Did we learn anything during covid which we can put to use after this which can give us better results in terms of managing RTC and RTI – example physical distancing, reducing exposure, lower ridership in vehicle etc. Effect of covid could be shift in travel mode to private vehicles or single rider vehicles to avoid crowd. Vehicle speed increases as more space with lower vehicle. There could be reductions in RTC, but RTI Fatal may not drop much.
8. Discussion para 3. USD123 million is for which year estimates? Interestingly 90% is based on indirect cost. Compared to other country studies, what are their range of % for indirect cost? Is it as high as this 90%?
9. What do the data indicate? RTC and RTI during covid should be compared during normal day within Nepal also. That is the right comparison and not against other countries like Fiji, Estonia, Cyprus etc.
10. Interesting to note on crashes involving ambulances during covid in comparison to normal day. Are they at more risk? If yes, what need to be done next? Increase in tractor usage does it adds to more crash? Any police data to show increase? Generally tractor as low speed vehicle pose less harm. Its only risk is when it shares the roadway with mixed traffic and the other traffic speed is very high and huge difference with the tractor speed.  
11. Conclusion – the content in conclusion is very much different compared to the text inside the article. Many aspects not touched in the article is being concluded here example of using PPE, safety helmet, seat belts and CRS. Prefer conclusion strictly based on this study findings.

Is the work clearly and accurately presented and does it cite the current literature?

Yes

If applicable, is the statistical analysis and its interpretation appropriate?

No

Are all the source data underlying the results available to ensure full reproducibility?

Partly

Is the study design appropriate and is the work technically sound?

Partly

Are the conclusions drawn adequately supported by the results?

No

Are sufficient details of methods and analysis provided to allow replication by others?

Partly

Reviewer Expertise:

Injury prevention and safety promotion

I confirm that I have read this submission and believe that I have an appropriate level of expertise to confirm that it is of an acceptable scientific standard, however I have significant reservations, as outlined above.

# Response

**Published:** 2020-11-26

**Authors:** Bhagabati Sedain

**Affiliations:** Tribhuvan University, Nepal

1.Abstract – For the methods sections, usage of secondary data from Police is fine and acceptable, but not from the media reports. Media reports does not cover all crashes and there could also be duplication of a single case is being reported by various media. Also, it’s the reporter and editor view. No one to verify it. Not recommended for scientific article unless no other options.

- Author response: Thank you for the concerns. We were aware that media reports do not cover all crashes, particularly those with non-fatal outcomes. During the period of lockdown and minimum vehicular movement, Nepali media covered 78% of all fatal incidents compared to police records (Table 1). We extracted detailed information for the individual crashes from media reports. A typical Nepali media report of a road crash, it reports – location of the crash; vehicle type and registration number; gender and age of victims (with names); outcomes of the crash. The details provided in the report obviously helped to see any duplication of the records. Such information was not possible to get from police records; the police source only provides compiled reports of incidents.
- We have revised the last sentence under "Methods" in the abstract.

2.Methods – is it possible to do away with the media reports and completely rely on the police data (secondary data)? What kind of information we can get from police? If that is sufficient, then go ahead with police data only.

- Author response: Using police data only will limit the information and we will not be able to make Figure 1 and Table 3, which is derived from media report data. We have amended the last sentence of the section Methods as – " *In this study, we have presented the results from the two data sources to provide the most possible details complemented by one-another."*

3.Results. Table 1. The paragraph tends to indicate as though the article is trying to compare two reporting system police vs media which is not the case as I believe you are using media to complement and close the gap for info not available from police source. Intention good, however the mechanism to address it is questionable since its unverified and its individually done by reporters.

- Author response: Thank you for the comment. We have revised our statement and removed the term 'compare' throughout the text because these two data sources complement one another. However, we have discussed the differences in the variables obtained from the two sources.

4.Table 2. Purpose of it? My understanding is based on the title, there could be two options of story flow: 1. Before and during covid: RTC and RTI status. Option 2: study RTC and RTI during covid (there must be a purpose and benefit of doing one). Till this stage not clear yet.

- Author response: Thank you for the suggestion. We described the data as "option 2" in your comment; the purpose was to show the districts that were the most affected. We have now revised the title of table 2 " *Table 2. Districts with the highest number of road traffic deaths in Nepal during national level COVID-19 lockdown*." We have replaced "Province 5" with its official name, Lumbini which is given after we submitted the manuscript.

5.Figure 1. Purpose of it? Does it tell us during covid the mapping of RTC and RTI are different compared to non-covid time? Therefore, this are the areas to be targeted and focused by the government towards addressing the problem. Is this what you intend to?

- Author response: The map (figure 1) shows the locations of road crashes that occurred during the lockdown. Therefore, it was not possible to compare the numbers before and during the lockdown. This information is derived from news reports, as the police data used in this study does not tell the location of crashes. We believe this map is a novel idea to show the locations of fatal road crashes and this would help relevant authorities to focus interventions.

6.Table 3. Purpose of it? Any different on its distribution / patterns from normal day which warrants attention? How best can we use this info towards our next move in terms of strategies or policy change with an aim to reduce RTC and RTI during covid.

- Author response: 1) the purpose of table 3 was to convey to the readers what type of vehicles were involved in road crashes; and the counterpart in case it was a collision. This information was extracted from media reports because this information was not available from police data used in this paper. We have made slight changes to the title of Table 3 as " *Distribution of road crashes during national level covid-19 lockdown according to the types of vehicles involved and their counterparts*." The key message here is – Motorcyclists, pedestrians and cyclists are the most at-risk road users from motorcyclists and the vehicles at essential services i.e. jeep/cars, tractors, trucks, and ambulances. Therefore, the enforcement of road safety laws must be of priority always.

7.Did we learn anything during covid which we can put to use after this which can give us better results in terms of managing RTC and RTI – example physical distancing, reducing exposure, lower ridership in vehicle etc. Effect of covid could be shift in travel mode to private vehicles or single rider vehicles to avoid crowd. Vehicle speed increases as more space with lower vehicle. There could be reductions in RTC, but RTI Fatal may not drop much.

- Author response: It is beyond the scope of this study, we only studied the period of lockdown where all non-essential travel was prohibited.

8.Discussion para 3. USD123 million is for which year estimates? Interestingly 90% is based on indirect cost. Compared to other country studies, what are their range of % for indirect cost? Is it as high as this 90%?

- Author response: Thanks for your comment. As mentioned in the reference cited in this paper, higher indirect costs are also seen in other countries. We have revised the sentences with additional reference: *"The total burden of road traffic injuries in Nepal is calculated to be approximately 123 million USD for 2017, and 90% of this amount comprised of indirect costs ( Banstola et al., 2020). The amount of indirect cost of road traffic injuries ranges from 51% in Iran to 90% in Nepal (Rezaei, Arab, Karami, & Akbari Sari, 2014; Banstola et al., 2020). This road crashes deaths toll demonstrated the economic impact of road crashes is bigger in Nepal."*

9.What do the data indicate? RTC and RTI during covid should be compared during normal day within Nepal also. That is the right comparison and not against other countries like Fiji, Estonia, Cyprus etc.

- Author response: Thank you very much for the comment. We have revised this section by adding the following text -

     " *This increased number of deaths and injuries during the lockdown in Nepal can be related to higher        speed due to lower traffic volume and limited law enforcement. High speed means higher impact if        there is a crash. It has also reported elsewhere that speed law violations and failing to stop due to          high speeding were increased during lockdown (Inada et al, 2020).*"

 10.Interesting to note on crashes involving ambulances during covid in comparison to normal day. Are they at more risk? If yes, what need to be done next? Increase in tractor usage does it adds to more crash? Any police data to show increase? Generally, tractor as low speed vehicles pose less harm. Its only risk is when it shares the roadway with mixed traffic and the other traffic speed is very high and huge difference with the tractor speed.  

- Author response: Thank you very much for the comment. a) Road crashes involving Ambulances are something that is not explored yet. Classification and standardization of ambulances in Nepal are yet evolving. Ambulances are a very important part of the post-crash response (pillar 5) aspect of road safety. Ambulances in Police records are not separately recorded and collectively included with other four-wheeled light vehicles (under jeep cars). Therefore, we cannot say whether there was an increase in ambulance movement or crashes during the COVID-19 lockdown. b) the use of tractors was increased for transportation of relief-aid materials (food, quarantine construction materials, etc) during the COVID-19 lockdown. It is true that the mix of vehicles with different speeds caused crashes; also, tractors from rural areas and hilly tracks were also reported.

11.Conclusion – the content in conclusion is very much different compared to the text inside the article. Many aspects not touched in the article is being concluded here example of using PPE, safety helmet, seat belts and CRS. Prefer conclusion strictly based on this study findings.

- Author response: Thank you very much for the comment and suggestions. We have now revised the conclusion section.
- Roadways are the major means of transport in Nepal. Just before this lockdown, a large number of people had to make journeys to their homes (mainly going out of Kathmandu and coming to Nepal from bordering India) by roads; many of those were exposed to the risks of unsafe roads transportation which led to their deaths and injuries. Our st udy found that deaths on Nepali roads was not stopped during lock down . Comparing the pattern of road cras hes during the same period last year, lock down witnessed almost half of the number of incidents (1 , 801 vs 3 , 480) and the number of vehicles involved in crashes (2 , 602 vs 5 , 560). When comparing the statistics with the situation with the three months before the lock down, it was observed that the percentages of tractors, trucks/tankers and cyclists was higher (published police records) . Therefore, this lockdown has reinforced how important the management of safer mobility issue is in Nepal. Interestingly, some of the preventative measures that have been proven effective to decelerate the spread of coronavirus apply in the context of road safety as these measures can teach us something for the road safety epidemic as well (Job, 2020). Therefore, for better road safety, unnecessary travels must be avoided and a safe distance should be maintained between the vehicles on the move. Likewise, the use of helmets, seat-belts, and child restraints are similar to the use of PPE, whereas the regular testing of vehicles, like COVID testing, is a must. In cities, traffic congestion was eased during the lockdown which consequently resulted higher speed, increasing the chances and impact of crashes. Therefore, awareness of safety and taking into account road and weather conditions when deciding to take a journey would help to keep people safe on the roads. Therefore, this lockdown has reinforced how important the management of safer mobility issue is in Nepal. Interestingly, some of the preventative measures that have been proven effective to decelerate the spread of coronavirus apply in the context of road safety as these measures can teach us something for the road safety epidemic as well ( Job, 2020 ). The Government of Nepal has mobilised unprecedented amount of resources in terms of human resources, budget and materials to address COVID-19 which has kept the rates of infection and deaths at minimum. If similar efforts and investments are done to address the problem of road traffic injuries, it would be possible to reverse the trend of ever-increasing burden of road injuries.
